# Large epigenome-wide association study identifies multiple novel differentially methylated CpG sites associated with suicidal thoughts and behaviors in veterans

**DOI:** 10.3389/fpsyt.2023.1145375

**Published:** 2023-05-25

**Authors:** Nathan A. Kimbrel, Melanie E. Garrett, Mariah K. Evans, Clara Mellows, Michelle F. Dennis, Lauren P. Hair, Michael A. Hauser, the VA Mid-Atlantic MIRECC Workgroup, Jean C. Beckham, Allison E. Ashley-Koch, Jean C. Beckham

**Affiliations:** ^1^Durham Veterans Affairs (VA) Health Care System, Durham, NC, United States; ^2^VA Mid-Atlantic Mental Illness Research, Education and Clinical Center, Durham, NC, United States; ^3^VA Health Services Research and Development Center of Innovation to Accelerate Discovery and Practice Transformation, Durham, NC, United States; ^4^Department of Psychiatry and Behavioral Sciences, Duke University School of Medicine, Durham, NC, United States; ^5^Duke Molecular Physiology Institute, Durham, NC, United States; ^6^Department of Psychology and Neuroscience, University of North Carolina at Chapel Hill, Chapel Hill, NC, United States

**Keywords:** suicide, epigenetics, methylation, psychiatry, suicidal ideation

## Abstract

**Introduction:**

The U.S. suicide mortality rate has steadily increased during the past two decades, particularly among military veterans; however, the epigenetic basis of suicidal thoughts and behaviors (STB) remains largely unknown.

**Methods:**

To address this issue, we conducted an epigenome-wide association study of DNA methylation (DNAm) of peripheral blood samples obtained from 2,712 U.S. military veterans.

**Results:**

Three DNAm probes were significantly associated with suicide attempts, surpassing the multiple testing threshold (FDR *q*-value <0.05), including cg13301722 on chromosome 7, which lies between the genes *SLC4A2* and *CDK5*; cg04724646 in *PDE3A*; and cg04999352 in *RARRES3.* cg13301722 was also found to be differentially methylated in the cerebral cortex of suicide decedents in a publicly-available dataset (*p* = 0.03). Trait enrichment analysis revealed that the CpG sites most strongly associated with STB in the present sample were also associated with smoking, alcohol consumption, maternal smoking, and maternal alcohol consumption, whereas pathway enrichment analysis revealed significant associations with circadian rhythm, adherens junction, insulin secretion, and RAP-1 signaling, each of which was recently associated with suicide attempts in a large, independent genome-wide association study of suicide attempts of veterans.

**Discussion:**

Taken together, the present findings suggest that *SLC4A2*, *CDK5*, *PDE3A*, and *RARRES3* may play a role in STB. CDK5, a member of the cyclin-dependent kinase family that is highly expressed in the brain and essential for learning and memory, appears to be a particularly promising candidate worthy of future study; however, additional work is still needed to replicate these finding in independent samples.

## 1. Introduction

The suicide mortality rate among U.S. military veterans has increased by 57% since 2001 (1) and is now 59% higher than the civilian rate (1). While a number of large-scale genome-wide association studies of suicidal thoughts and behaviors have been conducted in samples of both military veterans [e.g., (2–6)] and civilians [e.g., (7–11)], only a limited number of the genome-wide significant risk loci that have been identified by these studies have been successfully replicated in independent samples to date [e.g., (3, 7, 9, 11)].

There has also been considerable interest in the role that DNA methylation (DNAm) might play in the pathogenesis of suicidal thoughts and behaviors in recent years [e.g., (12–16)]. For example, a recent meta-analysis of DNAm data obtained from prefrontal cortex (*n* = 211) and cerebellum (*n* = 114) tissue revealed several epigenetic probes that were differentially methylated in suicide cases versus controls (16); however, as noted by Dada et al. (13) in their recent review of epigenetic studies of suicidal thoughts and behaviors: “Although sizable research has been carried out on this topic, most studies have been done on small-scale samples, and future research is required in larger samples with better clinical characterization of suicide phenotypes to investigate these epigenetic modifications further.”

Indeed, there have been only a few DNAm studies of suicidal thoughts and behaviors to date that have identified differentially methylated CpG sites that surpass the threshold for genome-wide significance [e.g., (14, 16)]. As a result, the genetic and epigenetic basis of suicidal thoughts and behaviors is largely unknown at the present time (12, 13, 15, 17). Further, while examination of brain methylation is critical for understanding the pathophysiology associated with suicidal thoughts and behaviors, examination of DNAm within peripheral blood samples offers the opportunity to develop biomarker assays that could be used in clinical settings to identify individuals at risk for attempting suicide. Accordingly, the objective of the present study was to advance this critical area of research by conducting the largest epigenome-wide association study of suicidal thoughts and behaviors to date utilizing peripheral blood samples obtained from a large and diverse cohort of U.S. military veterans.

## 2. Methods

### 2.1. Participants

The veteran participants included in the present analyses (*N* = 2,712) were drawn from the Post-Deployment Mental Health (PDMH) study and repository, a multi-site study of U.S. Afghanistan and Iraq era veterans (5, 18–20) conducted by the U.S. Department of Veterans Affairs (VA) Mid-Atlantic Mental Illness Research, Education, and Clinical Center [MIRECC; see (18) for detailed descriptions of study procedures]. Participants were recruited through mailings, advertisements, and clinician referrals from four VA hospitals located in the Southeastern U.S. The local institutional review board of each of the participating VA hospitals approved the study protocol, and written informed consent was obtained from all participants prior to enrollment in the PDMH study. After completion of the informed consent procedures, participants donated a blood sample and completed a battery of self-report questionnaires and interviews. To be eligible, participants had to have served in the U.S. military on or after September 11, 2001. Exclusion criteria included difficulty understanding the informed consent form or process, inability to travel to a data collection site, and/or lack of English fluency.

As can be seen in Table 1, the final sample of participants included in the present analyses (*N* = 2,712) was comprised of both non-Hispanic Black (NHB; 51.3%; *n* = 1,392) and non-Hispanic White (NHW; 48.7%; *n* = 1,320) veterans. Women veterans were also well-represented (25.3%; *n* = 685). Participants’ average age was 37.9 (SD = 10.3) years of age. Additional sample characteristics are provided in Table 1.

**Table 1:** Participant characteristics by race and ethnicity.

	Non-Hispanic black veterans (*N* = 1,392)		Non-Hispanic white veterans (*N* = 1,320)		
Variable	*N*	% / mean (SE)	*N*	% / mean (SE)	*p*-value
Age	1,392	39.35 (10.10)	1,320	36.31 (10.47)	<0.0001
Female	463	33.26%	222	16.82%	<0.0001
Current smoker	422	31.01%	365	27.97%	0.0857
Current PTSD	509	36.57%	485	36.74%	0.9241
BDI total score	1,339	14.98 (12.49)	1,301	15.26 (12.86)	0.6417
% Suicide attempt	46	5.46%	93	10.36%	0.0002
% Suicidal ideation	240	23.14%	312	27.93%	0.011

### 2.2. Clinical measures and phenotyping procedures

As in previous studies of suicide attempts conducted within the PDHM cohort [e.g., (5, 19, 20)], lifetime history of suicide attempts was coded as present if participants endorsed having attempted suicide one or more times on item #20 from the Beck Scale for Suicide Ideation [BSI; (21)]. In contrast, suicidal ideation was coded as present if participants endorsed any prior history of suicidal thoughts, plans, or attempts on items 1–20 on the BSI, item 9 on the Beck Depression Inventory-II (22), or item 15 from the Symptom Checklist-90 (23). As can be seen in Table 1, 5.1% (*n* = 139) of participants reported having attempted suicide at some point during their lifetime, whereas 20.4% (*n* = 552) of participants endorsed suicidal ideation. Participants were also assessed for posttraumatic stress disorder (PTSD) and other psychiatric disorders as part of the PDMH study procedures [e.g., (18, 19, 24)] with the Structured Clinical Interview for DSM-IV (25).

### 2.3. DNA methylation procedures

Whole blood samples were obtained via venipuncture. A total of 2,926 samples with sufficient DNA yield and quality were submitted for analysis on either the Infinium HumanMethylation450 Beadchip or the Infinium MethylationEPIC Beadchip. Samples were run separately by race/ethnicity, but randomized by sex and current PTSD status within each batch, except for one batch that included samples exclusively from male participants. Internal replicates were included and checked for consistency using single nucleotide polymorphisms (SNPs) that were incorporated on each array. Sample and probe quality control (QC) was performed using the minfi (26) and ChAMP (27) R packages. Samples were excluded based on the following QC metrics: average fluorescence signal intensity below 2,000 arbitrary units or < 50% of the mean intensity of all samples, >10% of probes were not detectable (detection value of *p* > 0.001), presence of a sex mismatch, or if the sample was deemed an outlier on principal component analysis plots. A total of 214 samples were removed as a result of the QC procedures, resulting in final sample of 2,712 samples that were available for the present analyses, including 1,320 samples from non-Hispanic White (NHW) and 1,392 samples from non-Hispanic Black (NHB) participants. Probe QC and data normalization was performed within each batch using the R package wateRmelon (28). Specifically, probes not detected (detection value of *p* > 0.001) in >10% of samples and those hybridizing to multiple locations in the genome were removed. Raw beta values were then normalized using the dasen approach (28) and adjustments for both batch and chip were accomplished using ComBat in the R package sva (29). M-values were calculated from the resulting normalized and adjusted beta values for statistical analysis.

### 2.4. Statistical approach

As can be seen in Table 1, participant characteristics were summarized by race/ethnicity using SAS v9.4 (SAS Institute, Cary, NC). Chi-square tests were used to test for differences between NHB and NHW participants on categorical variables, whereas linear regression was used to test for differences between NHB and NHW participants on age. Association analysis between each DNAm probe and suicide attempts and suicidal ideation was performed using MOMENT in the OSCA toolkit (30), controlling for batch and current smoking status. Specifically, linear mixed models with random effect components comprised of all other distal DNAm probes were used to account for unobserved confounders, including cell type composition, in a reference-free manner (30). Results were then meta-analyzed across the NHB and NHW samples using metal (31). A total of 423,945 overlapping CpG sites were analyzed in these analyses. False discovery rate (FDR) *q*-values were generated using the R package qvalue.^1^

The GEO2R tool^2^ was used to examine if the top blood-based DNAm probes associated with suicide attempts and ideation in the present study could be replicated in brain-based post-mortem studies of suicide and depression. Three publicly available datasets were utilized: GSE88890 (32), GSE137222/GSE137223 (16), and GSE41826 (33). GEO2R allows users to choose samples from publicly available data for downsteam differential analysis with limma (34, 35). For GSE88890, DNAm in cerebral cortex Brodmann area 11 (BA11) was compared in depressed individuals who died by suicide and non-psychiatric sudden death controls. The same comparison was made for DNAm from Brodmann area 25 (BA25). In GSE137222/GSE137223, we utilized DNAm from cerebellum in two cohorts of individuals who died by suicide and non-psychiatric sudden death controls. Finally, GSE41826 compared DNAm of post-mortem prefrontal cortex (PFC) fluorescence activated cell sorted (FACS) neuronal and glia cells from depressed individuals and matched control participants.

To better understand the degree to which our top blood-based DNAm probe values might correlate with brain-based methylation values at the same CpG site, the Iowa Methylation Array Graphing for Experimental Comparison of Peripheral tissue & Gray matter [IMAGE-CpG; (36)] database was used to look up blood–brain DNAm correlations for each of our top hits. The IMAGE-CpG database includes correlation coefficients and *p*-values for specific DNAm probes that were collected at the same time from both peripheral blood and live brain samples from patients with medically intractable epilepsy who were undergoing brain resection. As such, this database enables researchers to gain insight into the degree of blood–brain DNAm correlation at specific probe sites (36).

Finally, DNAm probes associated with suicide attempts or ideation (*p* < 0.001) were utilized in trait and pathway enrichment analysis within EWAS Toolkit.^3^ The largest overlapping set of DNAm probes in meta-analysis were included on the Infinium HumanMethylation450 Beadchip. Therefore, those probes were used as background for the enrichment analyses. Pearson correlations between DNAm of probes associated with suicide attempts and ideation in our dataset and gene expression in six tissues (brain, colon, kidney, liver, stomach, and testis) from EWAS datahub were calculated.

## 3. Results

Participant characteristics are summarized in Table 1. Across all participants (i.e., combining the non-Hispanic White and non-Hispanic Black participants), we observed that cases were slightly younger than controls. Specifically, suicide attempt cases were 34.2 years of age on average and suicidal ideation cases were 35.8 years of age on average, whereas control participants were 37.8 years of age on average. For both phenotypes, this difference was statistically significant (*p* < 0.001). With respect to differences by sex, we also observed that the percentage of females was higher in the NHB group compared to the NHW group (33.3% vs. 16.8%, *p* < 0.0001). NHB participants were also older (39.34 years vs. 36.3 years, *p* < 0.0001) than NHW participants. In contrast, NHW participants had a higher percentage of suicide attempts (10.4% vs. 5.5%, *p* = 0.0002) and reported more suicidal ideation (27.9% vs. 23.1%, *p* = 0.0110) compared to NHB participants in this study. NHB and NHW participants did not differ by current smoking status (*p* = 0.09), current PTSD status (*p* = 0.92), or total BDI score (*p* = 0.64).

**Figure 1:**
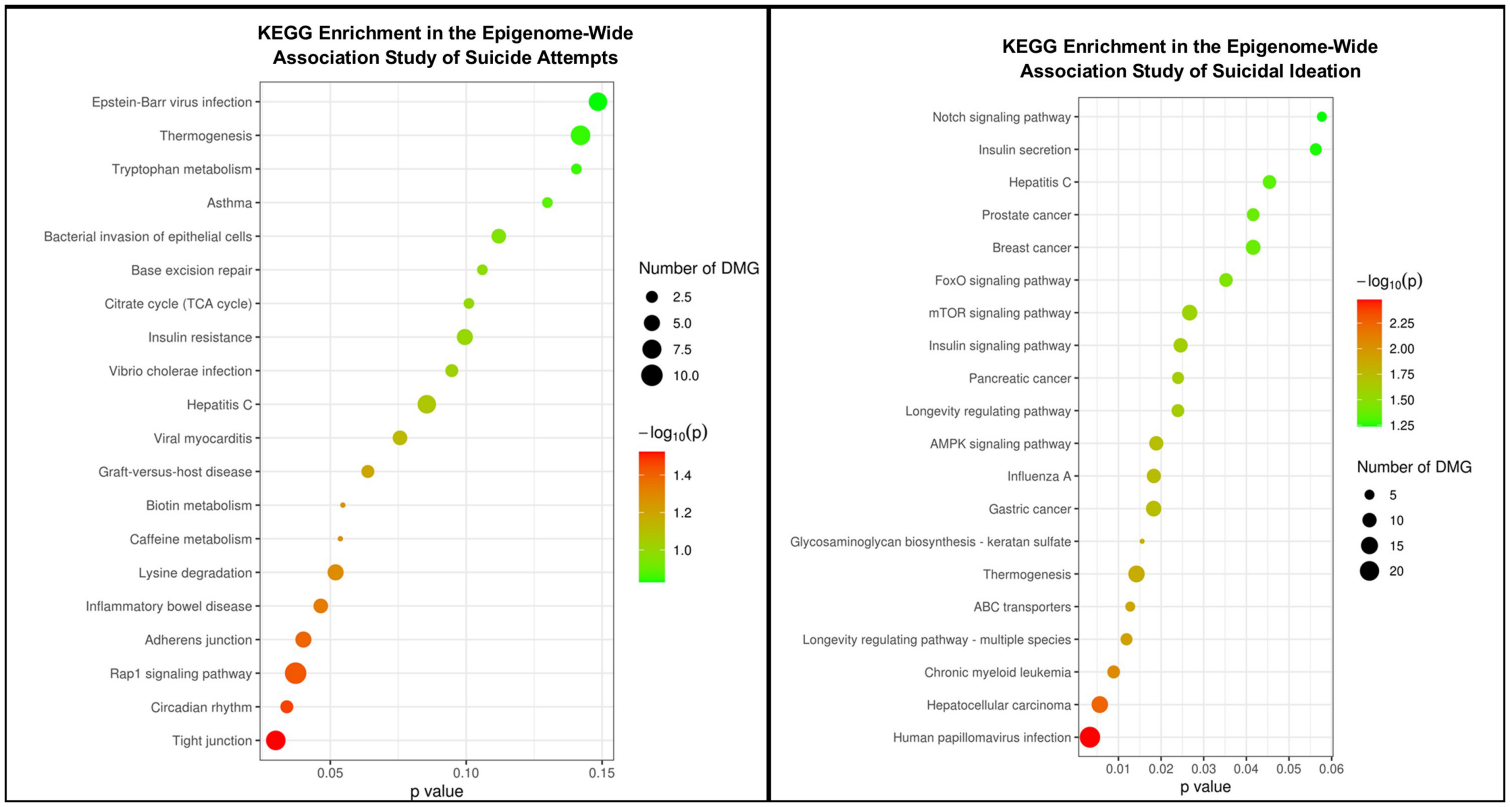
Summary of the pathway enrichment analyses for KEGG terms.

Three DNAm probes were significantly associated with suicide attempts in the present study, surpassing the multiple testing threshold (FDR *q*-value <0.05; Figure 1). cg13301722 on chromosome 7, which lies between *SLC4A2* and *CDK5*, was the CpG site most strongly associated with suicide attempts and was hypermethylated in the cases (*p* = 1.36×10^−8^, *q* = 0.0055). Two additional DNAm probes were also FDR significant: cg04724646 in PDE3A was hypomethylated in cases (*p* = 2.25×10^−7^, *q* = 0.0453) and cg04999352 in *RARRES3* was hypermethylated in cases (*p* = 3.59×10^−7^, *q* = 0.0482).

While no DNAm probes met strict FDR-significance in the suicidal ideation epigenome-wide association study, two DNAm probes were associated with suicidal ideation at FDR *q* < 0.10, including cg19789466 (*p* = 1.55×10^−7^, *q* = 0.0608), which is located in *OAS1* and was hypermethylated in suicidal ideation cases, and cg12161182 (*p* = 4.44×10^−7^, *q* = 0.0869), which is located in *NINJ2* and was hypomethylated in suicidal ideation cases.

Two of the DNAm probes that were associated with suicidal thoughts and behaviors in the present study were also found to be significantly associated with death by suicide and depression in two of the publicly-available datasets of post-mortem brain tissue we considered [i.e., (32, 33)]. Specifically, cg13301722 (*CDK5*/*SLC4A2*), which was associated with suicide attempts in our cohort, was found to also be differentially methylated in the cerebral cortex tissue (BA25) of suicide decedents compared with controls [*p* = 0.0254; (32)], whereas cg19789466 (*OAS1*), which was associated with suicidal ideation in our cohort, was found to be differentially methylated in prefrontal neuronal cells of depressed participants compared with controls [*p* = 0.0495; (33)].

Blood–brain DNAm correlations from the IMAGE-CpG database (36) were available for four of the top five associations identified in the present study, including cg13301722 (*SLC4A2/CDK5*: *r* = 0.31, *p* = 0.17); cg04999352 (*RARRES3*; *r* = 0.43, *p* = 0.05); cg1789466 (*OAS1*; *r* = 0.01, *p* = 0.96); and cg12161182 (*NINJ2*; *r* = 0.71, *p* = 0.0004).

Pathway enrichment analysis revealed associations between DNAm probes associated with suicide attempts in the present study and numerous KEGG terms (Figure 1), including circadian rhythm, adherens junction, insulin secretion, and RAP-1 signaling pathways. The latter associations are of particular interest, as each was also associated with the top associations identified in a recent independent, large-scale genome-wide association study of suicide attempts conducted among Veterans (4). Pathway enrichment analysis of DNAm probes associated with suicidal ideation also identified numerous KEGG terms, including AMPK signaling, insulin signaling, mTOR signaling, and numerous cancers (i.e., gastric, pancreatic, breast, and prostate; Figure 1).

**Figure 2:**
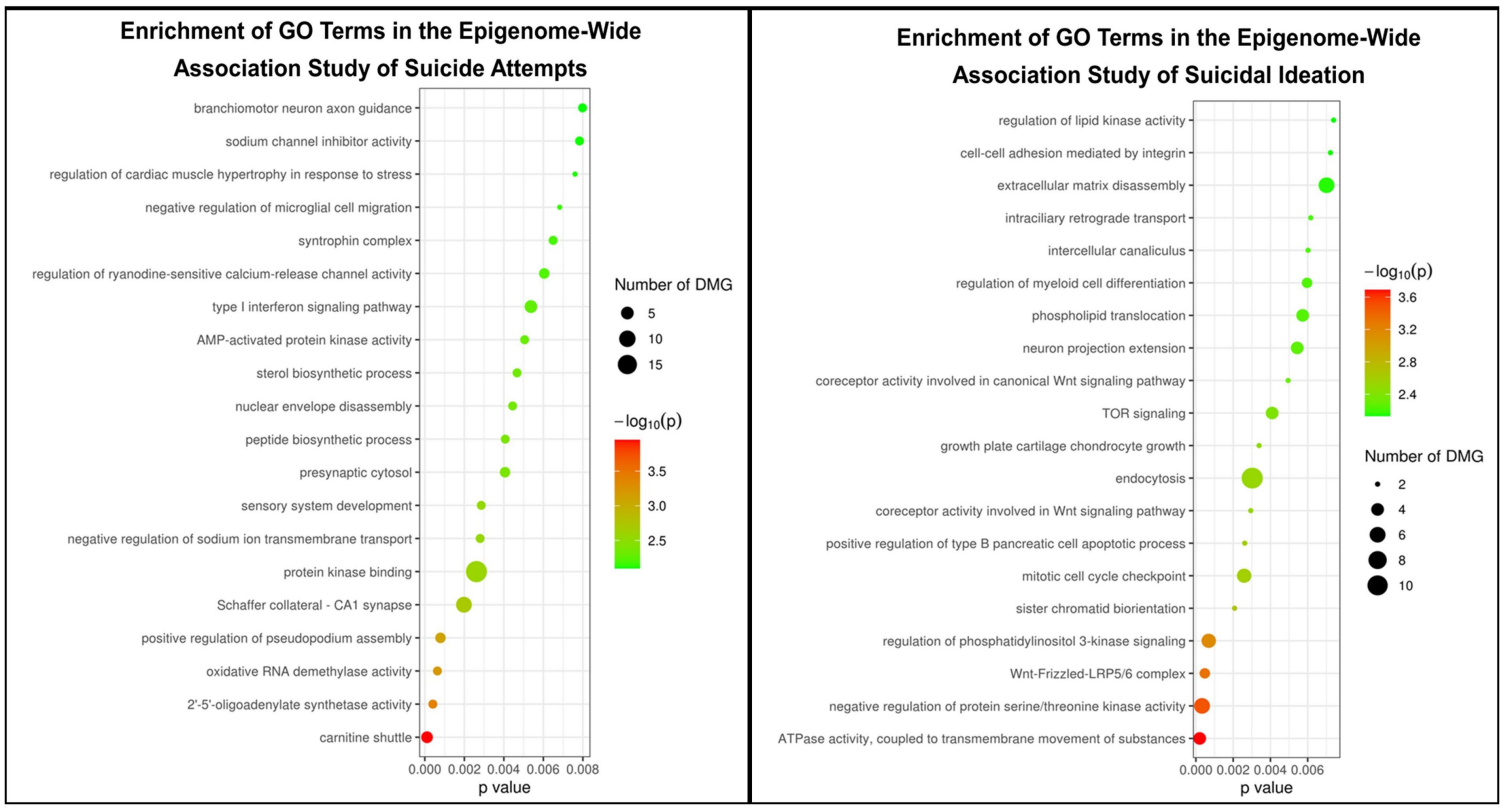
Summary of the pathway enrichment analyses for GO terms.

Pathway enrichment analysis of GO terms revealed associations between DNAm probes associated with suicide attempts in the present study and the type I interferon signaling and interferon-gamma-mediated signaling pathways, protein kinase binding, AMP-activated protein kinase activity, nucleotide-activated protein kinase complex, branchiomotor neuron axon guidance, and sodium channel inhibitor activity (Figure 2). Pathway enrichment analysis of GO terms associated with DNAm probes related to suicidal ideation in the present study identified terms associated with TOR signaling, WNT signaling, and neuron projection extension (among others; Figure 2).

Finally, trait enrichment analysis revealed that the DNAm probes associated with suicidal thoughts and behaviors in our sample were enriched for association with many other traits of interest. For example, as can be seen in Table 2, both suicide attempts and suicidal ideation were associated with substance use traits (e.g., smoking, alcohol consumption, substance use risk, and smoking cessation), major depression, childhood stress, maternal psychopathology and stress (e.g., maternal smoking, maternal alcohol consumption, maternal eating disorders, and maternal stress), socioeconomic traits (e.g., household socioeconomic status in childhood, poverty status, and educational attainment), as well as a variety of physical health conditions (e.g., cardiovascular risk, type 2 diabetes, obesity, lung cancer risk, asthma, and rheumatoid arthritis).

**Table 2:** Summary of the trait enrichment analyses.

Trait	Suicide attempt		Suicidal ideation	
	*p*	OR	*p*	OR
Aging	1.8E-38	3.9	6.4E-82	5.0
Asthma	1.6E-18	3.9	1.2E-26	3.9
Preterm birth	3.2E-15	4.2	4.1E-06	2.2
Smoking	2.5E-14	2.9	4.6E-09	2.1
Colorectal laterally spreading tumor	1.6E-12	4.4	1.3E-06	2.7
Papillary thyroid carcinoma	5.4E-12	5.0	1.6E-14	4.6
Follicular thyroid carcinoma	1.8E-11	5.1	3.8E-08	3.5
Infertility	3.2E-09	4.6	2.5E-12	4.5
Hepatocellular carcinoma (HCC)	1.6E-08	3.5	2.6E-03	1.9
Mixed connective tissue disease (MCTD)	4.7E-08	33.3	1.6E-04	15.4
Household socioeconomic status in childhood	3.5E-07	12.9	4.7E-06	9.0
Rheumatoid arthritis (RA)	2.2E-06	3.9	1.5E-03	2.4
Puberty	8.0E-06	13.5	4.2E-07	12.6
Atopy	1.1E-05	3.2	3.2E-07	3.2
Alcohol consumption	2.4E-05	5.5	2.1E-08	6.2
Kabuki syndrome (KS)	3.3E-05	5.3	7.2E-03	2.9
Maternal smoking	9.8E-05	2.9	1.6E-04	2.5
Type 2 diabetes (T2D)	3.2E-04	2.8	5.5E-07	3.2
Prostate cancer	4.9E-04	2.5	4.6E-03	2.0
B Acute lymphoblastic leukemia with t(12;21)(p13.2;q22.1); ETV6-RUNX1	5.3E-04	2.9	2.2E-03	2.3
Behcet’s disease	2.2E-03	2.7	5.8E-03	2.2
Obesity	2.4E-03	2.2	1.3E-08	3.1
Primary Sjögren’s Syndrome (pSS)	3.9E-03	2.8	8.7E-04	2.8
Maternal alcohol consumption	6.4E-03	3.0	6.1E-03	2.6
SETD1B-related syndrome	7.6E-03	2.9		
Bronchodilator response	8.0E-03	15.3		
Educational attainment	1.2E-18	148.9		
Smoking cessation	1.0E-14	17.1		
HIV frailty	1.0E-13	44.5		
Lung function	3.8E-13	78.9		
Oral squamous cell carcinoma (OSCC)	1.2E-11	3.0		
Adenoma	9.8E-11	4.0		
IgG glycosylation	1.3E-10	789.4		
Cardiovascular risk	2.2E-10	654.2		
Lung cancer risk	3.2E-09	280.9		
Metabolic trait	2.4E-08	158.1		
Down syndrome	1.2E-07	2.5		
Preeclampsia	1.9E-07	6.1		
Mortality	2.6E-07	4.6		
Lung carcinoma	4.3E-07	73.5		
Neurodevelopmental presentations and congenital anomalies (ND/CAs)	3.7E-06	9.3		
Urological cancer	1.1E-05	31.5		
Trihalomethanes (THM) exposure	1.5E-05	28.7		
Soluble tumor necrosis factor receptor 2 (sTNFR2) levels in plasma	3.1E-05	23.9		
Hyperdiploid B acute lymphoblastic leukemia	4.3E-05	4.3		
Perinatal polychlorinated biphenyls and polychlorinated dibenzofurans exposure	2.1E-04	104.0		
Crohn’s disease (CD)	5.2E-04	3.7		
Sedentary behavior	6.0E-04	60.0		
Autoimmune diseases	6.3E-04	10.7		
Cognitive function	7.5E-04	10.2		
Sex-related differences in chronic lymphocytic leukemia	7.9E-04	5.7		
High saturated fatty acids diet	1.1E-03	4.0		
Psoriasis	1.4E-03	2.9		
Substance-use risk	2.1E-03	30.9		
Plasma homocysteine (Hcy) levels	3.1E-03	25.4		
Poverty status	3.1E-03	25.1		
Vitamin B12 supplement	3.3E-03	6.8		
Recurrent stroke	3.5E-03	23.8		
B Acute lymphoblastic leukemia with dic(9;20)(p11;q13)	3.5E-03	3.3		
Orofacial cleft			1.1E-07	15.1
Werner syndrome			1.1E-07	7.7
Atherosclerosis			2.8E-07	5.1
Metabolic syndrome (MetS)			1.7E-06	10.4
Childhood stress			2.0E-06	10.2
Maternal eating disorders			2.1E-05	460.7
Food allergy			4.5E-05	21.7
Incident myocardial infarction occurrence			9.3E-05	172.4
Atypical antipsychotic-induced insulin resistance			1.1E-04	153.8
Sexual functioning			1.1E-04	153.8
Klinefelter syndrome			1.5E-04	15.7
Claes-Jensen syndrome			1.9E-04	5.3
Osteoporosis			1.9E-04	115.4
Prenatal perfluoroalkyl substance exposure			2.9E-04	7.0
Coffin-Siris syndrome (CSS)			7.0E-04	10.4
Placental microbes			5.2E-03	3.1
Major depression disorder			6.9E-03	5.5
Recurrent stroke			6.9E-03	16.7
Maternal stress			7.5E-03	15.9
Epithelial ovarian cancer risk			7.7E-03	15.7
Hormone therapy			7.7E-03	5.3
Hyperdiploid B acute lymphoblastic leukemia			8.9E-03	2.5

## 4. Discussion

The present analyses represent the largest epigenome-wide association study of suicidal thoughts and behaviors to date and identified several interesting candidate genes. The DNAm probe most strongly associated with suicide attempts in the present study lies near *CDK5* (cg13301722; *p* = 1.36×10^−8^, *q* = 0.0055), a member of the cyclin-dependent kinase family that is highly expressed in human brain tissue. Notably, cg13301722 was also found to be differentially methylated in the cerebral cortex tissue (BA25) of suicide decedents compared with controls (*p* = 0.0254) in a publicly available dataset from a previous study (32), providing further evidence that this CpG site may be associated with risk for suicidal thoughts and behaviors.

Within adult brains, CDK5 has been shown to regulate neuronal growth, axonal formation, synaptic plasticity, synaptic functioning, learning and memory formation, neuronal survival, pain signaling, drug addiction, circadian clock regulation, and long-term behavioral changes (37). CDK5 dysregulation has also been associated with multiple neurodegenerative diseases, including Alzheimer’s disease, Parkinson’s disease, and Huntington’s disease (37–39). In addition, genome-wide association studies have found associations between *CDK5* and numerous psychiatric phenotypes of interest, including schizophrenia, depression, attention-deficit hyperactivity disorder, sleep difficulties, chronotype, and smoking cessation (40), whereas previous epigenome-wide associations studies have identified associations between *CDK5* and schizophrenia, smoking, and maternal smoking (41). To our knowledge, the present findings represent the first documented association between *CDK5* and suicidal thoughts and behaviors; however, a previous post-mortem study did report significant differences in CDK5 expression in the prefrontal cortex of individuals who died by suicide compared with control participants who died by other causes (42).

Two additional DNAm probes were also FDR-significant in the epigenome-wide association study of suicide attempts: cg04724646 in *PDE3A* (*p* = 2.25×10^−7^, *q* = 0.0453) and cg04999352 in *RARRES3* (*p* = 3.59×10^−7^, *q* = 0.0482). PDE3A is a protein coding member of the cyclic nucleotide phosphodiesterase family which is responsible for hydrolyzing both cAMP and cGMP, whereas *RARRES3* is a retinoic acid receptor responder that has been characterized as a tumor suppressor (43). Previous genome-wide association studies have associated *PDE3A* with schizophrenia, alcohol dependence, cannabis use, smoking, neuroticism, risk taking, and lithium response in bipolar patients (40), whereas *RARRES3* has been associated with alcohol consumption, irritability, seeking professional help for mental distress, and number of cigarettes smoked per day (40). To our knowledge, the present findings represent the first documented association between *RARRES3, PDE3A,* and suicidal thoughts and behaviors.

While no DNAm probes met strict FDR-significance in the suicidal ideation epigenome-wide association study, two DNAm probes were associated with suicidal ideation at FDR *q* < 0.10, including cg19789466 (*OAS1; p* = 1.55×10^−7^, *q* = 0.0608) and cg12161182 (*NINJ2; p* = 4.44×10^−7^, *q* = 0.0869). *OAS1* is an interferon-induced gene that plays a critical role in innate cellular antiviral responses. Previous genome-wide association studies have identified associations between *OAS1* and neuroticism, risk taking, depression, and Parkinson’s disease (40). cg19789466 (*OAS1*) was also observed to be differentially methylated in the prefrontal neuronal cells of depressed participants compared with controls (*p* = 0.0495) in a publicly-available dataset from a previous study [i.e., (33)], providing further evidence that this CpG site may be associated with risk for depression and/or suicidal thoughts and behaviors. *NINJ2* is a cell surface adhesion protein that is highly expressed in brain tissue and helps to promote axonal growth and recovery from nerve injuries. Previous genome-wide association studies have identified associations between *NINJ2* and schizophrenia, bipolar disorder, obsessive–compulsive disorder, extraversion, and chronotype (40), whereas previous epigenome-wide association studies have identified associations between *NINJ2* and smoking, alcohol consumption, maternal alcohol consumption, and fetal alcohol syndrome. *NINJ2* was also recently associated with suicidal behavior in an Iranian sample (44).

With respect to the enrichment analyses, it is noteworthy that several of the strongest pathway enrichments observed in the present study were also observed in a recent large-scale genome-wide association study of suicide attempts conducted in an independent sample of veterans, including the circadian rhythm, adherens junction, insulin secretion, and RAP-1 signaling pathways (4). The observed enrichments for branchiomotor neuron axon guidance, neuron projection extension, mTOR signaling, and WNT signaling are also of interest, given prior research in this area [e.g., (3, 45, 46)]. Specifically, axon guidance was recently identified as the top overall KEGG pathway associated with suicidal thoughts and behaviors following a meta-analysis across four ancestries in the largest genome-wide association study of suicidal thoughts and behaviors conducted among veterans to date (3). In addition, Niculescu et al. (46) have previously identified mTOR signaling as a top pathway in a multi-cohort study of gene expression in relation to suicidal thoughts and behaviors. A prior study of gene expression associated with suicide attempts among U.S. military veterans also identified the mTOR and WNT signaling pathways (45). Moreover, mTOR signaling plays a key role in the rapid anti-depressant and anti-suicidal effects associated with ketamine (45, 46), whereas lithium—a drug for the treatment of bipolar disorder that also shows promise for suicide prevention [e.g., (47)]—regulates WNT signaling (45, 46).

Finally, the associations between the top DNAm CpG sites associated with suicidal thoughts and behavior in the present study and smoking, alcohol consumption, substance use risk, and major depression are also of great interest, given that each of these traits has been prospectively associated with increased risk for attempting suicide in prior research [e.g., (48)]. The associations with numerous adverse childhood experiences and stressors (e.g., childhood stress, poverty status, household socioeconomic status in childhood, maternal stress, maternal smoking, maternal alcohol consumption, and maternal eating disorders) are also notable, as these types of stressful experiences would be expected to increase risk for suicidal thoughts and behaviors as well as psychopathology, more generally, potentially through their effects on DNAm and other forms of epigenetic modification [e.g., (49, 50)]; however, more work and more complex research designs (e.g., longitudinal studies of high-risk families) are necessary to elucidate the complex relationships between parental psychopathology, stressful childhood experiences, DNAm, and future risk for suicidal thoughts and behaviors.

### 4.1. Study limitations

The findings from the present study should be interpreted within the context of several limitations. First, the HumanMethylation450 and MethylationEPIC Beadchips utilized in the current study only assess a small fraction of the human epigenome. As a result, there are many additional CpGs that could be associated with suicidal thoughts and behaviors that were not assessed in the present study. Second, while the present work represents the largest EWAS of suicidal thoughts and behaviors to date, the sample size (*N* = 2,712) utilized in the current study is not sufficiently large to detect the small effect sizes commonly observed among genetic and epigenetic studies of psychiatric conditions. Thus, future work utilizing larger sample sizes and/or meta-analytic techniques are clearly needed. Third, the methylation data utilized in the present study was blood-based. While examination of DNAm markers within peripheral blood samples offers the opportunity to develop biomarker assays that could be used in clinical settings to identify individuals at risk for attempting suicide, large brain-based DNAm studies are still needed to facilitate understanding of the pathophysiology that underlies suicidal thoughts and behaviors. Finally, while a significant strength of the present analyses was the inclusion of a large cohort of NHB Veterans (*n* = 1,392), additional work is still needed to examine the role of DNAm in relation to suicidal thoughts and behaviors in other non-NHW ancestral groups. A closely-related concern relates to the fact that the present cohort was also predominantly male. Thus, additional work is also needed to study the association between DNAm and suicidal thoughts and behaviors in larger samples that have substantially greater representation of females to ensure that findings are broadly generalizable and to facilitate sex-specific analyses. Given that there are well-established differences in rates of suicidal thoughts and behaviors as a function of sex [e.g., (3, 48)], such analyses are likely to be of high importance for this particular phenotype.

### 4.2. Conclusion

Taken together, the present findings suggest that CDK5, a member of the cyclin-dependent kinase family that is highly expressed in the brain and implicated in regulating neuronal growth, axonal formation, synaptic plasticity, learning and memory formation, drug addiction, circadian clock regulation, neurodegenerative disorders, and long-term behavioral changes, may also play a role in the pathogenesis of suicidal thoughts and behaviors. We also identified numerous pathway enrichments of interest, including the circadian rhythm, adherens junction, insulin secretion, RAP-1 signaling, mTOR signaling, and WNT signaling pathways, all of which have been previously associated with suicidal thoughts and behaviors in prior studies [e.g., (4, 45, 46)]. Finally, we observed enrichment for numerous traits of interest, including established risk factors for suicidal thoughts and behaviors [e.g., smoking, alcohol consumption, substance use risk, and major depression; (48)] as well as a variety of adverse childhood experiences (e.g., childhood stress, poverty status, household socioeconomic status in childhood, maternal stress, maternal smoking, maternal alcohol consumption, and maternal eating disorders) that could potentially influence risk for suicidal thoughts and behaviors through DNAm and other forms of epigenetic modification [e.g., (49, 50)]. Collectively, our findings point to the potential of eventually developing blood-based epigenetic biomarker tests capable of accurately identifying individuals at risk for suicidal thoughts and behaviors; however, additional studies with larger sample sizes and more complex research designs will be necessary to make this vision a reality.

## Data availability statement

The datasets presented in this article are not readily available because Durham VA IRB requirements governing the sharing of genetic and epigenetic data do not allow for sharing of individual-level data to individuals who are not IRB-approved members of the study team. Requests to access the datasets should be directed to angela.kirby@va.gov.

## Ethics statement

The studies involving human participants were reviewed and approved by Durham Veterans Affairs Health Care System IRB. The patients/participants provided their written informed consent to participate in this study.

## Author contributions

NK, MG, MH, AA-K, MD, and JB contributed to the conception and design of the study. NK, MG, and AA-K were responsible for developing the statistical plan. MG and AA-K conducted the statistical analyses. NK and MG wrote the first complete draft of the manuscript. MKE, CM, MFD, and LPH assisted NK with manuscript preparation. All authors contributed to the article and approved the submitted version.

## The VA Mid-Atlantic MIRECC Workgroup

The VA Mid-Atlantic MIRECC Workgroup contributors for this paper include: Jean C. Beckham, PhD, Patrick S. Calhoun, PhD, Eric Dedert, PhD, Eric B. Elbogen, PhD, John A. Fairbank, PhD, Robin A. Hurley, MD, Jason D. Kilts, PhD, Nathan A. Kimbrel, PhD, Angela Kirby, MS, Sarah L. Martindale, PhD, Christine E. Marx, MD, MS, Scott D. McDonald, PhD, Scott D. Moore, MD, PhD, Rajendra A. Morey, MD, MS, Jennifer C. Naylor, PhD, Jared Rowland, PhD, Robert D. Shura, PsyD, Cindy Swinkels, PhD, Larry A. Tupler, PhD, Elizabeth E. Van Voorhees, PhD, and Ruth Yoash-Gantz, PsyD.

## Funding

This research was supported by Award #IK2CX000525 to Dr. Kimbrel from the Clinical Science Research and Development (CSR&D) Service of the Department of Veterans Affairs (VA) Office of Research and Development (ORD), Award #I01BX002577 to Dr. Beckham from the Biomedical Laboratory Research and Development (BLRD) Service of VA ORD, and a Senior Research Career Scientist Award (#lK6BX003777) to Dr. Beckham from CSR&D. The authors also received support from the VA Mid-Atlantic Mental Illness Research, Education and Clinical Center (MIRECC), the Mental Health and Research Services of the Durham VA Healthcare System, the Department of Psychiatry and Behavioral Sciences at the Duke University School of Medicine, and the Duke Molecular Physiology Institute. The views expressed in this article are those of the authors and do not necessarily reflect the position or policy of the VA, the US government, Duke University, or any other affiliated institution.

## Conflict of interest

The authors declare that the research was conducted in the absence of any commercial or financial relationships that could be construed as a potential conflict of interest.

## Publisher’s note

All claims expressed in this article are solely those of the authors and do not necessarily represent those of their affiliated organizations, or those of the publisher, the editors and the reviewers. Any product that may be evaluated in this article, or claim that may be made by its manufacturer, is not guaranteed or endorsed by the publisher.

## Footnotes

^1^https://github.com/StoreyLab/qvalue

^2^https://www.ncbi.nlm.nih.gov/geo/geo2r/

^3^https://ngdc.cncb.ac.cn/ewas/toolkit
